# Evolution of Dysphagia Rehabilitation in Japan Since the 1980s: Expanding Dental Roles in Interprofessional Care—A Narrative Review

**DOI:** 10.3390/healthcare14081060

**Published:** 2026-04-16

**Authors:** Mika Miyaoka, Kosuke Muraoka, Shuji Awano, Wataru Fujii

**Affiliations:** 1 Oral Rehabilitation Center, Kyushu Dental University Hospital, Kitakyushu 803-8580, Japan; r15fujii@fa.kyu-dent.ac.jp; 2 Division of Clinical Education Development and Research, Department of Oral Function, Faculty of Dentistry, Kyushu Dental University, Kitakyushu 803-8580, Japan; muraoka@kyu-dent.ac.jp (K.M.); awa-shu@kyu-dent.ac.jp (S.A.); 3 Division of Dysphagia Rehabilitation, Faculty of Dentistry, Kyushu Dental University, Kitakyushu 803-8580, Japan

**Keywords:** dysphagia rehabilitation, population aging, long-term care insurance, community-based integrated care system, dental professionals, transdisciplinary collaboration, home-based care, triad model

## Abstract

**Background/Objectives**: Japan, the world’s first super-aged society, has confronted rapid population aging and increasing healthcare demands earlier than any other country. In this context, dysphagia rehabilitation has become a critical issue affecting quality of life and survival. With nearly 30% of the population aged ≥65 years, Japan has developed a distinctive dysphagia rehabilitation model characterized by interprofessional collaboration and dental involvement. This narrative review describes its historical evolution and structural characteristics. **Methods**: This narrative review employed a structured literature search of PubMed and Ichushi-Web, supplemented by manual searches of policy documents and professional guidelines. Publications from 1980 to January 2026 were included if they addressed dysphagia rehabilitation systems or dental involvement in Japan. Both English- and Japanese-language sources were analyzed using thematic synthesis. **Results**: Japan’s dysphagia rehabilitation model evolved alongside population aging and is embedded within the universal health insurance and long-term care insurance systems. A prominent characteristic is the sustained involvement of dental professionals, who contributed to the foundational development of the field and remain actively involved across care settings, particularly within community- and home-based care. The system is further supported by certification frameworks, a triadic model integrating rehabilitation, nutrition, and oral health, and institutionalized interprofessional education. **Conclusions**: Previous studies have examined specific aspects of dysphagia care in Japan, but few have examined the overall structure of the system. This review maps the fundamental structure of Japan’s dysphagia rehabilitation model within its historical and policy context, offering insights relevant to dysphagia care in other aging societies.

## 1. Introduction

Dysphagia, defined as difficulty or discomfort in swallowing, affects individuals across the lifespan and healthcare settings. It arises from a wide range of conditions and is most commonly associated with neuromuscular diseases, stroke, dementia, cancer and its treatments, respiratory disorders, congenital conditions, and age-related physiological decline [1]. Beyond its physical manifestations, dysphagia profoundly compromises quality of life (QoL). Eating, a fundamental human activity and social practice, may lose its pleasure and become distressing or dangerous; in severe cases, even swallowing saliva can pose a life-threatening risk. In addition, dysphagia often necessitates dietary restrictions and is frequently accompanied by fear of choking, which can lead to social withdrawal, frustration, and depression [2]. Among older adults, dysphagia often coexists with sarcopenia and malnutrition, forming a vicious cycle that can result in serious medical conditions, such as dehydration, aspiration pneumonia, and even death.

Dysphagia and its complications not only jeopardize personal health but are also recognized as a substantial contributor to healthcare burden, being associated with prolonged hospital stays and increased healthcare costs, partly due to increased demand for hospital care, long-term care services, and nutritional or respiratory support [3]. Within the International Classification of Functioning, Disability and Health (ICF) framework, the World Health Organization (WHO) conceptualizes dysphagia as a condition associated with increased morbidity, mortality, and treatment costs [4,5].

As populations age, the prevalence of dysphagia is rising sharply. A meta-analysis reveals it affects approximately 30% of community-dwelling older adults, nearly 50% of hospitalized geriatric patients, and more than half of nursing home residents [6]. It is also widespread among patients with stroke, Alzheimer’s disease, Parkinson’s disease, and cancer. As these conditions become more prevalent with population aging, dysphagia—one of their common associated complications—is expected to increase in parallel. Moreover, dysphagia is frequently underdiagnosed, in part because a substantial proportion of individuals experience “silent aspiration” without overt symptoms, leading to delayed recognition until serious complications arise across diverse care settings [7,8,9,10]. As life expectancy increases globally, the number of individuals at risk of dysphagia is expected to grow steadily, particularly in Asia—the fastest-aging region in the world.

Japan has reached the most advanced stage of demographic aging. As the world’s first super-aged society, Japan has one of the highest proportions of adults aged ≥65 years— approximately 29.3% in 2024 [11], projected to reach 34.8% by 2040 [12]—as well as one of the longest life expectancies globally. In Japan, aspiration pneumonia—one of the most common complications of dysphagia among older adults—represents the sixth leading cause of death and accounts for 86.7% of pneumonia cases in individuals aged ≥70 years [13,14,15]. This growing clinical burden has intensified pressure on Japan’s medical and long-term care systems, while increasing demand for effective dysphagia management within an already resource-constrained healthcare environment.

Over the past four decades, the development of Japan’s dysphagia rehabilitation has closely paralleled the country’s demographic and policy transition from an aging society (1970–1994), to an aged society (1994–2007), and finally to a super-aged society (2007–present). This parallel evolution reflects a bidirectional and mutually reinforcing dynamic between healthcare policy reforms and the evolution of Japan’s dysphagia rehabilitation system. Successive reforms, including the establishment of the long-term care insurance system (LTCIS), a publicly funded system providing comprehensive care services for older adults requiring long-term care, have substantially expanded the accessibility and delivery of dysphagia-related services. In turn, advances in dysphagia management have been regarded as potentially contributing to the alleviation of healthcare burden, for example, by reducing the incidence of complications such as aspiration pneumonia and supporting functional recovery [16,17].

Historically, dysphagia rehabilitation in Japan was initially developed within the fields of rehabilitation medicine and otolaryngology. Over time, however, the field has evolved into a highly collaborative, interprofessional system of care involving dentists, dental hygienists (DHs), nurses, speech–language–hearing therapists (SLHTs), dietitians, care managers, and other allied health professionals. In this expanding framework, dental professionals have played a distinctive role [18,19,20], broadening their scope from oral hygiene and routine dental treatment to active participation in swallowing evaluation and dysphagia management as oral health specialists in interprofessional teams. This long-standing dental involvement is considered to have contributed to the development of a more coordinated care framework integrating rehabilitation, nutrition support, and oral management. This integrated approach has been increasingly recognized as a defining feature of contemporary dysphagia practice in Japan, particularly in the care of older adults [21,22]. The prior establishment of formal certification systems for dysphagia nursing and advanced dental hygienists [23,24], along with additional certification pathways for related professions, has provided structural support for this evolving model.

These developments have shaped a distinctive and mature dysphagia rehabilitation model in Japan, developed over more than four decades in response to the demands of an aging population. As both a pioneer in recognizing dysphagia as a major aging-related health issue and a forerunner in developing system-level responses, Japan’s experience may offer a potentially valuable reference for other countries confronting similar demographic challenges.

Although previous studies have described specific aspects of dysphagia rehabilitation in Japan, such as clinical practices, professional roles, and selected components of interdisciplinary care, these investigations have largely focused on individual elements rather than the system as a whole. As a result, a comprehensive understanding of the system-level development and structure of dysphagia rehabilitation in Japan—particularly in relation to policy context, interprofessional collaboration, and the integration of dental professionals—remains limited. This limitation also constrains the transferability of Japan’s experience to other aging societies, underscoring the need for a more comprehensive system-level analysis.

To address this gap, this narrative review examines the dysphagia rehabilitation model in Japan within its historical and policy context, focusing on its system structure, clinical practices, and the expanding role of dental professionals within collaborative care frameworks. By focusing on the Japanese experience, this review aims to provide an in-depth understanding of this distinctive model rather than providing cross-national comparisons. At the same time, the insights derived from this context have broader international relevance. Japan’s experience illustrates how dysphagia rehabilitation can be developed as a system-level, integrated domain in response to rapid population aging, offering transferable lessons for healthcare systems facing similar demographic transitions, as well as for those seeking to develop more integrated and interprofessional models of care. In particular, the integration of policy frameworks and the expanded role of dental professionals can inform the design and optimization of dysphagia care in other healthcare systems.

## 2. Methods

### 2.1. Study Design and Review Approach

This study was conducted as a narrative review aimed at synthesizing the historical development, policy context, and interprofessional structure of dysphagia rehabilitation in Japan. Given the aim of providing a contextualized and historically grounded overview of system development and professional role integration—rather than quantitatively evaluating clinical interventions or outcomes—and considering the heterogeneity of included sources (e.g., diverse study designs, data types, and policy-oriented materials), many of which are not designed for outcome-based comparison and therefore do not allow for meaningful quantitative synthesis, a narrative review approach was considered more appropriate than a systematic review or meta-analysis.

### 2.2. Search Strategy

To enhance transparency and reproducibility, a structured literature search was conducted using PubMed and Ichushi-Web (Japan Medical Abstracts Society), a major Japanese medical literature database.

The search combined keywords related to dysphagia and the Japanese context. Both English and Japanese search terms were used, including representative terms such as “dysphagia,” “deglutition disorders,” “swallowing rehabilitation,” “Japan,” “oral management,” “dental involvement,” and “interprofessional care,” as well as their Japanese equivalents. These terms were combined using Boolean operators (e.g., AND, OR) to identify relevant literature across clinical, policy, and interprofessional domains.

The search included publications available up to January 2026.

### 2.3. Study Selection and Eligibility Criteria

Literature sources included peer-reviewed journal articles, books, national surveys, and official policy documents published between 1980 and 2026. From a methodological perspective, this timeframe was selected to reflect the period during which dysphagia rehabilitation emerged and developed as a structured and interprofessional clinical field in Japan. Previous studies have similarly described this development as largely occurring since the 1980s [19,20]. While earlier studies related to swallowing disorders exist, they were often fragmented, lacked standardized diagnostic frameworks, and were not directly comparable with contemporary frameworks of dysphagia rehabilitation.

Publications were included if they met one or more of the following criteria:

(1) Addressed dysphagia rehabilitation practices or systems in Japan;
(2) Discussed the role of dental professionals or collaborative care practices;
(3) Described policy frameworks, clinical guidelines, or healthcare system structures relevant to dysphagia care.

Both English- and Japanese-language sources were included to ensure comprehensive coverage of both international and domestic evidence. This approach was particularly important given that a substantial proportion of relevant literature—including policy documents, clinical guidelines, and practice-based reports—is published primarily in Japanese and may not be fully captured through English-language databases alone.

Publications were excluded if they focused solely on highly specific clinical interventions without relevance to system-level understanding, or if they lacked sufficient methodological or contextual detail. Therefore, a linguistically inclusive and context-sensitive search strategy was adopted to better capture the institutional and practice-based dimensions of dysphagia rehabilitation in Japan.

### 2.4. Data Sources for Policy and Guidelines

In addition to database searches, relevant policy documents, clinical guidelines, and reports from professional organizations—including the Japan Society of Dysphagia Rehabilitation (JSDR), the Japanese Geriatrics Society, and the Japanese Society of Gerodontology—as well as materials from the Ministry of Health, Labour and Welfare (MHLW), were identified through manual searches of official websites and reference lists of included articles.

All retrieved literature sources were screened in a stepwise manner based on titles, abstracts, and full-text review for thematic relevance to the study objectives. The screening and selection process was conducted by the authors based on predefined eligibility criteria. In total, more than 80 sources were included in the final synthesis, encompassing peer-reviewed articles, policy documents, and organizational reports.

### 2.5. Data Synthesis

Given the narrative nature of this review, a formal systematic review framework and quantitative synthesis were not employed. Instead, selected sources were qualitatively and thematically analyzed, with particular attention to historical evolution, institutional frameworks, and professional role development. Findings were synthesized to construct a system-level overview of dysphagia rehabilitation in Japan.

### 2.6. Methodological Limitations

As a narrative review, this study is subject to inherent limitations, including potential selection bias and the absence of formal quality assessment of included sources. Although efforts were made to enhance transparency and comprehensiveness, the synthesis may reflect the authors’ interpretation of the available literature.

## 3. Historical Evolution of Dysphagia Rehabilitation in Japan

To understand Japan’s current dysphagia rehabilitation model, it is essential to examine the unique historical and structural characteristics that shaped its development. The field has evolved through the interplay of demographic pressures, healthcare policy, and interprofessional clinical practice.

### 3.1. The Japanese Concept of Dysphagia: “Sesshoku–Enge”

**Figure 1:**
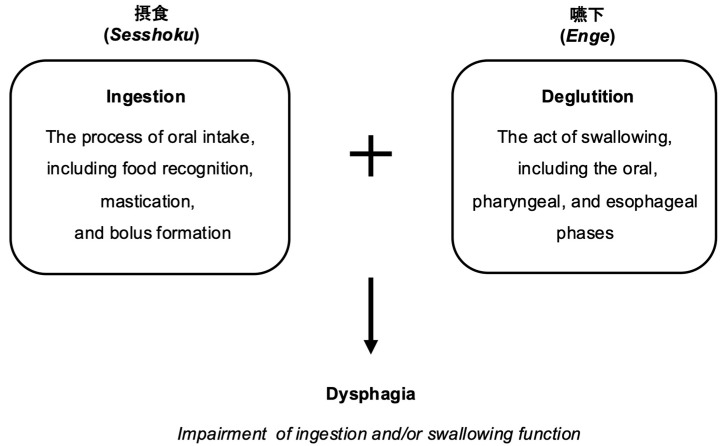
Conceptual definition of dysphagia as “*Sesshoku–Enge*” in the Japanese context. The kanji term *Sesshoku–Enge* (摂食嚥下) represents dysphagia as a functional continuum encompassing ingestion (*sesshoku*) and deglutition (*enge*), reflecting the integrated swallowing process from oral intake to esophageal transport.

Unlike many medical terms of Western origin that are typically transliterated into katakana in Japanese medical usage, dysphagia was instead defined in kanji as 摂食嚥下障害 (*Sesshoku–Enge shōgai*, literally “ingestion–deglutition disorder”). *Sesshoku* refers to ingestion, whereas *Enge* denotes deglutition; together, these two kanji terms conceptualize dysphagia as a functional disorder encompassing the entire process from oral intake to esophageal passage [18] (Figure 1).

This conceptual clarity, established from the outset, contributed to the establishment of dysphagia as a coherent discipline situated at the intersection of otolaryngology, dentistry, oral surgery, gastroenterology, rehabilitation medicine, nursing, and nutrition. The kanji-based definition helped reduce ambiguity in both clinical practice and scholarly discourse, thereby facilitating the early development of Japan’s dysphagia rehabilitation framework.

Moreover, because kanji convey meaning on a conceptual rather than purely phonetic level, the term is intuitively accessible even to non-professionals, potentially improving communication with patients and caregivers and enhancing public awareness of dysphagia as a health condition.

### 3.2. Development of Japan’s Dysphagia Rehabilitation in Parallel with Aging-Related Health and Long-Term Care Policies

**Figure 2:**
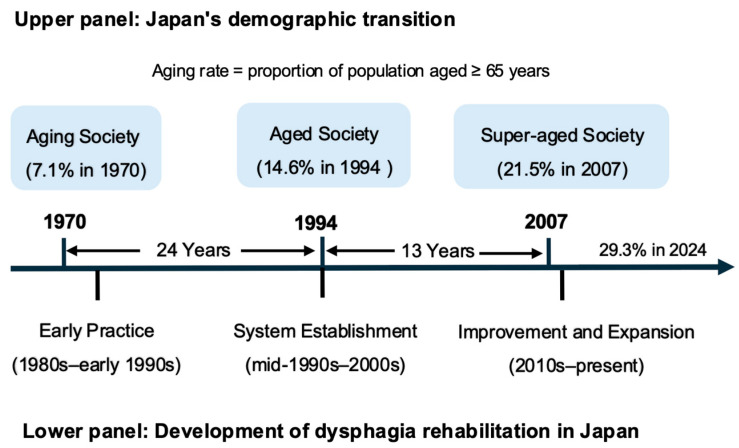
Timeline depicting Japan’s demographic transition (upper panel) and the parallel development of dysphagia rehabilitation (lower panel). The upper panel presents the proportion of the population aged ≥65 years, illustrating the transitions to an aging society (1970), an aged society (1994), and a super-aged society (2007), with the aging rate reaching 29.3% in 2024. The intervals between these milestones—24 years from aging to aged and 13 years from aged to super-aged—highlight the accelerating pace of population aging. The lower panel outlines major phases in the evolution of dysphagia rehabilitation in Japan.

From a demographic perspective, Japan’s demographic aging is conventionally classified according to the proportion of individuals aged ≥65 years: an aging society (≥7%), an aged society (≥14%), and a super-aged society (≥21%), reached in 1970, 1994, and 2007, respectively [25], as shown in Figure 2. According to the Cabinet Office of Japan, the country has remained in the super-aged stage since 2007, with a continued increase in the proportion of the old-old population (≥75 years). This rapid demographic transition fundamentally reshaped Japan’s health and long-term care systems, creating institutional and policy conditions that facilitated the expansion of dysphagia rehabilitation services.

**Figure 3:**
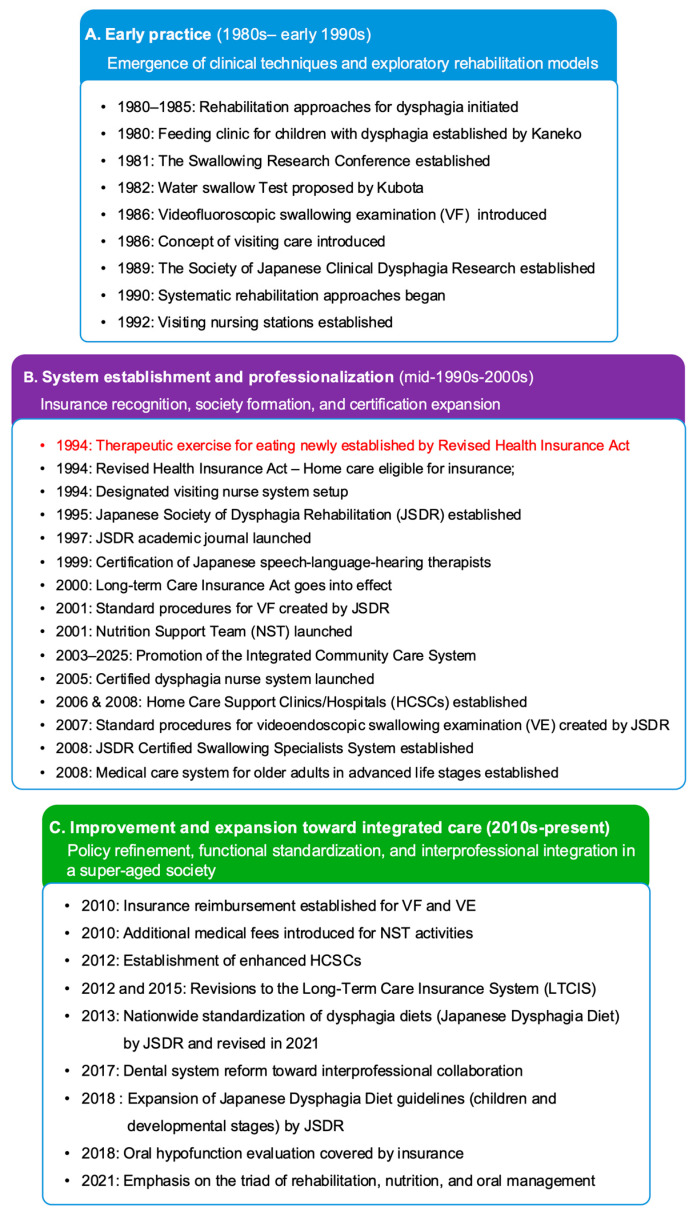
Three developmental stages of dysphagia rehabilitation in Japan in parallel with societal aging. Panel (**A**) illustrates the early practice phase (1980s–early 1990s), characterized by the emergence of rehabilitation-oriented concepts and foundational clinical practices. Panel (**B**) illustrates the phase of system establishment and professionalization (mid-1990s–2000s), characterized by policy legitimation, the formation of professional societies, the expansion of certification systems, and the refinement of clinical practices. Panel (**C**) illustrates the phase of expansion toward integrated care (2010s–present), characterized by policy reforms, expanded insurance coverage, and the consolidation of interprofessional, community-based care. Major policy-driven milestones in dysphagia rehabilitation are highlighted in red.

The major milestones in the development of dysphagia rehabilitation in Japan are summarized in Figure 3. As illustrated in Figure 2 and Figure 3, discussions on dysphagia rehabilitation began to emerge in the 1980s; a system establishment phase followed in the mid-1990s; and further refinement and expansion accelerated in the late 2000s, corresponding to Japan’s deepening super-aged demographic structure.

Throughout these phases, a wide range of healthcare professionals—including physicians, nurses, SLHTs, and dentists—contributed to the progressive codification of clinical practice, the establishment of professional societies and certification systems, and the incorporation of dysphagia management into reimbursement and long-term care policy frameworks.

#### 3.2.1. Early Practice (1980s–Early 1990s)

During this exploratory phase, pioneering clinical efforts gradually crystallized, forming the initial foundation for systematic dysphagia rehabilitation in Japan (Figure 3A). In 1980, a feeding clinic for children with dysphagia was established under the leadership of pediatric dentist Dr. Yoshihiro Kaneko, who later played an important role in extending dysphagia rehabilitation concepts to adult populations.

By the early 1990s, several hospitals had begun implementing structured dysphagia rehabilitation approaches, reflecting an early transition from isolated, case-based practice to more organized clinical interventions [26]. In parallel, new assessment methods were introduced into clinical settings, including the water swallow test (a screening method widely used in Japan) in 1982 and videofluoroscopic swallowing examination (VF), an instrumental assessment used to evaluate swallowing function in 1986 [26]. Early clinical reports documented their initial application and clinical utility, providing an empirical basis for later standardization.

These clinical developments occurred alongside early aging-related policy reforms that promoted community- and home-based care. For example, the introduction of the visiting care concept in 1986 [27] and the subsequent establishment of visiting nurse stations in 1992 signaled a gradual shift in care delivery away from hospital-centered models [28].

These early clinical innovations and policy initiatives together established the conceptual and organizational groundwork for the subsequent institutionalization of dysphagia rehabilitation.

#### 3.2.2. System Establishment and Professionalization (Mid-1990s–2000s)

With Japan’s transition to an aged society in 1994, dysphagia rehabilitation reached a major institutional turning point (Figure 3B). That year, “therapeutic exercise for eating” was newly established as a reimbursable category under the national health insurance system, thereby formally recognizing dysphagia rehabilitation as a legitimate component of medical care. This policy milestone marked the transition of dysphagia rehabilitation from exploratory, case-based clinical efforts to a standardized and institutionally supported field of medical practice.

In 1995, the JSDR was founded, representing the first formal interdisciplinary academic society in Japan dedicated specifically to dysphagia care. By bringing together physicians, dentists, SLHTs, nurses, dietitians, and other allied professionals, the JSDR consolidated interprofessional collaboration as a defining structural feature of the emerging field. Its establishment accelerated the formalization of professional training and certification pathways, beginning with the introduction of national certification for SLHTs in 1997 [29], followed by certification programs for dysphagia nurses (2005) [23,30], swallowing specialists (2008) [31], and advanced dental hygienists (2008) [24]. These initiatives strengthened professional identity, clarified role differentiation, and enhanced workforce capacity in dysphagia rehabilitation. Concurrently, the JSDR pioneered the standardization of diagnostic procedures. It published the first national standardized protocol for VF examination in 2001 [32], followed by the first standardized protocol for videoendoscopic examination of swallowing (VE), an instrumental assessment used to assess pharyngeal swallowing and airway protection in 2007 [33]. These efforts marked Japan’s earliest attempts to unify dysphagia assessment and establish nationally consistent clinical standards.

Over time, these organizational and professional developments laid the foundation for the sustained expansion of the society and the broader professional community. As of 2025, JSDR membership exceeds 15,000 practitioners from multiple disciplines, with SLHTs accounting for approximately 31% and dentists accounting for around 19%, followed by nutritionists, nurses, dental hygienists, and physicians [31]. This growth reflects the continued integration of multiple professional disciplines and the increasing organizational maturity of dysphagia rehabilitation in Japan.

During the same period, the accelerating aging population prompted continuous policy responses that further embedded dysphagia rehabilitation within national health and long-term care systems. A central milestone was the launch of the LTCIS in 2000, through which dysphagia and aspiration pneumonia were incorporated into the Kihon Checklist (basic checklist) to facilitate early detection of functional decline and inform care-level determination [34,35]. From 2006 onward, the LTCIS introduced dedicated categories of oral intake-related services, covering interventions aimed at improving oral function, maintaining oral ingestion, and supporting transitions from tube feeding to oral intake across different care settings [36].

Nationwide initiatives also reinforced the expansion of community- and home-based care during this phase. These included revisions to the Health Insurance Act in 1994, which extended insurance coverage to home-based medical care [27], and established a designated visiting nurse service system [28], the introduction of Nutrition Support Teams (NSTs) in 2001—multidisciplinary teams typically comprising physicians, nurses, dietitians, and pharmacists to optimize nutritional management in clinical settings [37], and the progressive development of the Community-Based Integrated Care System (CBICS) from 2003, when a government research report first articulated the need for an integrated community care framework in long-term care policy [38]. This was followed by the establishment of Home Care Support Clinics and Hospitals (HCSCs) between 2006 and 2008 [39], as well as the introduction of a new medical care system for the elderly in the latter stage of life in 2008 [40].

As a result, the clinical systematization and policy reforms of this period established the structural foundation for Japan’s modern dysphagia rehabilitation model and prepared the system for further consolidation in the super-aged era.

Following the system establishment phase in the mid-1990s, further refinement and expansion accelerated in the late 2000s, corresponding to Japan’s deepening super-aged demographic structure.

#### 3.2.3. Improvement and Expansion Toward Integrated Care (2010s–Present)

At this stage of demographic transition, the pace of demographic change accelerated markedly. Whereas the transition from an aging society (1970) to an aged society (1994) took 24 years, the shift to super-aged status occurred within only 13 years (2007). This rapid demographic transition intensified pressure on both healthcare and long-term care systems, placing comparable demands on dysphagia rehabilitation practices. In response, the field faced increasing structural and clinical challenges while continuing to refine and expand established practices (Figure 3C).

Several key challenges emerged during this period in both clinical practice and policy discussions, including:

- Increased clinical demand for VF and VE examinations;
- A growing need for systematic evaluation of oral hypofunction, defined as a decline in multiple oral functions rather than impairment in a single domain (a concept specific to the Japanese healthcare system);
- The absence of a unified dysphagia diet classification, leading to inconsistencies across care settings.

In response to these emerging challenges, several clinical and system-level measures were implemented. New medical fee structures for VE and VF examinations were established in 2010 [41], and diagnostic evaluation of oral hypofunction was initially incorporated into insurance coverage in 2018 [42]. In addition, dysphagia diets were standardized nationwide through the introduction of the Japanese Dysphagia Diet in 2013, followed by expanded guidelines in 2018 addressing children and individuals in developmental stages [43,44].

In parallel, reforms within the broader aging-related policy landscape further reinforced the maturation of Japan’s dysphagia rehabilitation model. These included the introduction of additional medical fees for NST activities in 2010 [41], the introduction of an enhanced HCSC designation in 2012 [27], and revisions to the LTCIS in 2012 and 2015, which introduced new requirements for multidisciplinary feeding management and daily dietary observation [36].

During the same period, dentistry in Japan underwent a major shift toward treatment and long-term management, with increasing integration into interdisciplinary care in response to increasing age-related care demands [45,46]. Furthermore, academic societies expanded certification pathways to foster advanced expertise and strengthen professional capacity, including the establishment of the Certified Dentist in Dysphagia Rehabilitation in 2014 [47], followed by the Certified Swallowing Consultation Physician/Dentist and Certified Swallowing Consultant credentials in 2018 [48].

More recently, Japan has increasingly promoted a triad model integrating rehabilitation, nutrition, and oral management as a central strategy to enhance functional recovery and overall outcomes in its rapidly aging population [49].

Collectively, these clinical, policy, and educational developments improved care transitions across acute, convalescent, home, and facility-based settings, reinforced coordinated interprofessional practice, and consolidated a patient-centered and scalable dysphagia rehabilitation model adapted to evolving demographic and clinical demands. While these system-level transformations were shaped by contributions from multiple healthcare professions, dentistry has played a particularly distinctive and enduring role in the development of Japan’s dysphagia rehabilitation model. The historical origins and evolving functions of dental professionals therefore warrant dedicated discussion in the following section.

### 3.3. Dentists as Pioneers in Shaping Dysphagia Rehabilitation in Japan

Among the diverse professionals involved in Japan’s contemporary dysphagia rehabilitation system, dentists have played a historically significant role in shaping early interdisciplinary pathways.

From the early developmental stages, the active involvement of dentists became a defining feature of Japan’s interprofessional dysphagia rehabilitation model. Unlike in many countries, where dental practice is largely confined to oral care and routine dental treatment, Japanese dentists are formally positioned within the healthcare system as core contributors to dysphagia rehabilitation, particularly in the preparatory (oral) and pharyngeal phases of swallowing. In routine clinical practice, they are permitted to conduct both VF and VE examinations, a feature that distinguishes the Japanese model from those of many other countries.

A pivotal figure in the development of dentistry’s role in dysphagia rehabilitation was Dr. Yoshihiro Kaneko, a pediatric dentist who made substantial and formative contributions to the field. Beginning his pioneering work in 1977, and drawing on observations of clinical models abroad, Dr. Kaneko established one of the earliest dedicated feeding clinics for children with dysphagia in 1980. Through clinical practice, he identified fragmented disciplinary organization as a key structural barrier to the early development of dysphagia care in Japan. Although otolaryngology, dentistry, oral surgery, and gastroenterology were all involved, each discipline operated within a narrow, discipline-specific scope. This compartmentalized structure impeded the recognition of dysphagia as a systemic, function-oriented clinical condition at that time [18,50].

Key contributions led by Dr. Kaneko and collaborating dental professionals included the following:

- Disciplinarization (1987–2005): Publication of foundational textbooks on adult and pediatric dysphagia rehabilitation, together with public lecture programs addressing the absence of structured dysphagia education in medical and dental curricula [18];
- Insurance recognition (1994): Approval of therapeutic exercises for eating under dental social insurance, representing the first formal integration of rehabilitation services into the dental reimbursement framework, with subsequent extension to medical insurance [18,19];
- Dental leadership in the formation of JSDR (1995): Building on earlier academic exchanges in Japan—such as the Swallowing Research Conference and the Society of Japanese Clinical Dysphagia Research—dental leaders played a central role in the establishment of the JSDR in 1995. Dr. Kaneko later served as its inaugural president (1997–2005), further consolidating dentistry’s leadership in the emerging interdisciplinary field. To promote academic exchange and the dissemination of clinical knowledge, the society launched *The Japanese Journal of Dysphagia Rehabilitation* in 1997 [16,24]. This interdisciplinary foundation was later complemented by contributions from other disciplines, including the publication of clinical practice guidelines for dysphagia by the Japanese Society of Otorhinolaryngology in the late 2000s [51].

Today, dental professionals are actively involved across a broad spectrum of dysphagia-related practice. Their roles encompass oral function and swallowing assessments, identification and management of oral dysfunction, implementation of direct and indirect rehabilitation interventions, caregiver and family education, and support for safe and efficient feeding practices [52]. These professional activities extend across care environments, including acute-care hospitals—such as participation in the NSTs [53], convalescent rehabilitation wards [54], long-term care facilities, hospitals without dental departments, and settings in the community and home, where visiting dental services play a critical role in maintaining continuity of dysphagia management across care transitions [55,56].

Moreover, Japan’s high dentist density (84.2 per 100,000 population) [57], together with the active involvement of local dental associations in organizing community-based dental services, continuing professional education, and home-visit programs across the lifespan [46], provides a strong structural foundation for the implementation of dysphagia rehabilitation. This workforce and organizational capacity further reinforce dentistry’s pivotal role within Japan’s interprofessional dysphagia rehabilitation system.

Collectively, these historical, institutional, and clinical developments positioned dental professionals as structurally embedded contributors within Japan’s interprofessional dysphagia rehabilitation system, integrating oral management into coordinated, patient-centered care for an aging society.

## 4. Current Model of Dysphagia Rehabilitation in Japan

Japan’s dysphagia rehabilitation system has evolved into an integrated, multi-tiered model designed to accommodate the heterogeneous and dynamic nature of swallowing disorders, which may be acute or chronic, intermittent or progressive, and vary across patients or disease stages. To address these diverse clinical trajectories, Japan emphasizes coordinated vertical and horizontal collaboration across its extensive medical, dental, and long-term care resources.

Within the Universal Healthcare System (UHS), the LTCIS, and CBICS, patients are able to access timely diagnosis, continuous rehabilitation, and smoother transitions among acute, convalescent, long-term, and home-based care. This coordinated structure has been associated with improved continuity of care and is intended to reduce the risk of severe complications—such as aspiration pneumonia and potentially avoidable hospitalizations, thereby alleviating the overall healthcare burden.

At the core of this model are two interrelated strategies:

(1) Leveraging the high density of Japan’s medical and dental facilities to enhance accessibility and continuity of care;
(2) Strengthening transdisciplinary collaboration to improve care coordination, efficiency, and resource utilization.

Through this framework, earlier identification and continuous management of swallowing dysfunction are promoted, care transitions are more effectively coordinated, and patient-centered care is more consistently achieved. Although regional variation and facility-specific sub-models exist, the system as a whole is characterized by clear role delineation, close inter-facility coordination, and flexible team-based practice.

### 4.1. Patient Characteristics and Care Settings

Dysphagia in Japan is distributed across multiple levels of the healthcare and long-term care system, reflecting its high prevalence in both medical and care settings. National surveys indicate that approximately 13.6% of acute-care inpatients and 31.6% of patients in convalescent rehabilitation wards present with dysphagia, while more than half of residents in long-term care facilities exhibit swallowing impairment [58].

Importantly, dysphagia is not confined to institutional populations. Analyses of Japan’s Long-Term Care Insurance nationwide claims dataset indicate that swallowing and feeding difficulties are also prevalent among older adults receiving services in community- and home-based settings [36]. Community-based epidemiologic studies further report that a substantial proportion of middle-aged and older adults screen positive for swallowing difficulty using questionnaire-based or functional screening tools [59,60].

Taken together, these findings suggest that swallowing dysfunction in Japan should be conceptualized as a continuum spanning acute hospitals, rehabilitation facilities, institutional long-term care, and community-dwelling populations. Accordingly, dysphagia rehabilitation is required across multiple tiers of care, extending well beyond inpatient settings. With the continued expansion of visiting nursing services and home-visit medical and dental care, an increasing proportion of dysphagia management is expected to occur outside hospitals, underscoring the growing importance of coordinated community-based care in Japan’s super-aging society.

### 4.2. Healthcare Resources and Their Roles in Dysphagia Rehabilitation

#### 4.2.1. Characteristics of Japan’s Healthcare Infrastructure

**Figure 4:**
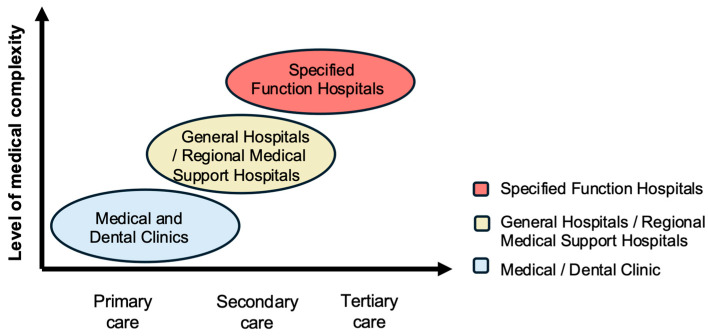
Distribution of medical facilities across Japan’s three-tier healthcare system. Medical and dental clinics mainly provide primary care; general hospitals and regional medical support hospitals operate predominantly at the secondary level; and specified function hospitals deliver tertiary care. The vertical axis denotes the level of medical complexity, and the horizontal axis represents the three-tier structure of healthcare provision.

Japan maintains an exceptionally dense and geographically well-distributed healthcare infrastructure, forming a critical structural foundation for dysphagia rehabilitation practice in Japan. According to the Medical Facility Dynamic Survey published by the MHLW, Japan has approximately 8000 hospitals, 105,000 medical clinics, and 65,000 dental clinics nationwide, reflecting extensive spatial coverage of healthcare services [61]. In the Japanese healthcare system, hospitals are generally defined as institutions with more than 20 beds, including national, public, and university hospitals, whereas smaller facilities are categorized as medical or dental clinics. Together, these facilities form a multi-layered network ranging from primary care clinics to highly specialized hospitals (Figure 4).

The high spatial density of healthcare facilities contributes to substantial geographic accessibility, including for older adults with mobility limitations. In addition, patient survey data indicate relatively short waiting times for consultations, suggesting timely access at the point of care [62].

Beyond physical proximity, Japan’s healthcare accessibility is also supported by national health and long-term care policies that promote continuity of care across different settings [63] (see Section 4.3). Within this system, dysphagia rehabilitation services can be delivered not only in hospital environments but also in outpatient clinics, long-term care facilities, and patients’ homes.

Collectively, this combination of dense infrastructure, geographic accessibility, institutional support, and timely service delivery facilitates early detection, prompt intervention, and continuous management of dysphagia across disease stages.

#### 4.2.2. Roles of Healthcare Facilities in Dysphagia Rehabilitation

To promote the efficient allocation and coordination of regional medical resources, Japan’s medical system is organized into three levels: primary, secondary, and tertiary care [64] (Figure 4).

1. Primary care (medical and dental clinics)
  - Medical clinics

Medical clinics often serve as the first point of contact for patients with suspected dysphagia, particularly in the acute or early stages. Physicians—including primary care doctors, rehabilitation physicians, and otorhinolaryngologists—are primarily responsible for initial screening, etiological assessment, and basic management of mild cases. Depending on institutional capacity, care is provided through outpatient consultations and, when necessary, home-visit services. In this setting, portable VE examination systems enable bedside evaluation and timely interprofessional decision making.

Importantly, when dysphagia is closely related to oral conditions, or when specialized oral assessment and intervention are required, patients are frequently referred to dental clinics for further evaluation and management. This referral pathway reflects the functional integration of medical and dental services within Japan’s primary care–based dysphagia rehabilitation framework.

  b. Dental clinics

Within primary care, dental clinics play a distinct and specialized role in Japan’s dysphagia rehabilitation system, reflecting both the close physiological relationship between oral function and swallowing and the systemic demands of a rapidly aging society. In routine clinical practice, physicians—including internists and otorhinolaryngologists—frequently seek dental consultation or refer patients to dental clinics when dysphagia is closely associated with oral conditions or when continuous management through home-visit medical services is not feasible.

From a policy perspective, a major turning point occurred in 2017, when the MHLW formally redefined the role of dentistry within the national health insurance system—from a treatment-centered discipline to one encompassing prevention, long-term management, and active participation in integrated care pathways [65]. This policy shift emphasized community-based oral healthcare delivered by family dentists and explicitly positioned dental professionals within integrated care pathways. Following this reform, visiting dental services expanded substantially: by 2017, approximately 13,000 dental clinics—out of roughly 68,000 nationwide—were providing home-visit dental care, a number projected to double by 2040 [46]. In parallel, a certification system for comprehensive family dentist offices was introduced in 2016 within the framework of reimbursement reform, enabling certified clinics to claim additional management fees and further reinforcing dentists’ roles within the architecture of community-based healthcare delivery [66].

These institutional reforms have not only reshaped the payment landscape but have also stimulated professional discourse on the future organization of oral and dysphagia care. More recently, the Japanese Dental Science Federation has proposed the concept of “1.5-tier dental clinics,” envisioned as community-based, multifunctional facilities bridging primary dental care and hospital dentistry. This model aims to strengthen regional dental care capacity by supporting home-visit services and providing specialized care for medically complex patients in an aging population with complex care needs [67].

**Figure 5:**
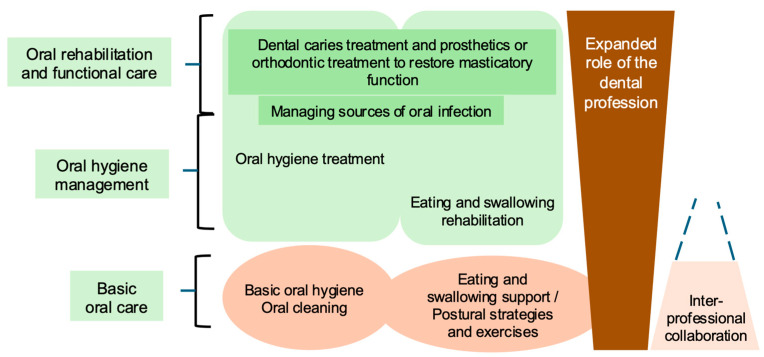
Conceptual framework of expanded oral health care and the evolving role of the dental profession in Japan. This figure illustrates an expanded conceptualization of oral health care in Japan that extends beyond conventional oral hygiene to encompass oral rehabilitation, functional care, and training for eating and swallowing. Within this framework, the dental profession plays a central role not only in managing dental disease and sources of oral infection, but also in supporting masticatory function and interprofessional care across medical and long-term care settings. The figure highlights a continuum from basic oral care to advanced oral health care, accompanied by increasing collaboration with other health and care professionals. Concept adapted from [46]. Colors and dashed lines are used to indicate different levels of care and the expansion of interprofessional collaboration, respectively.

**Figure 6:**
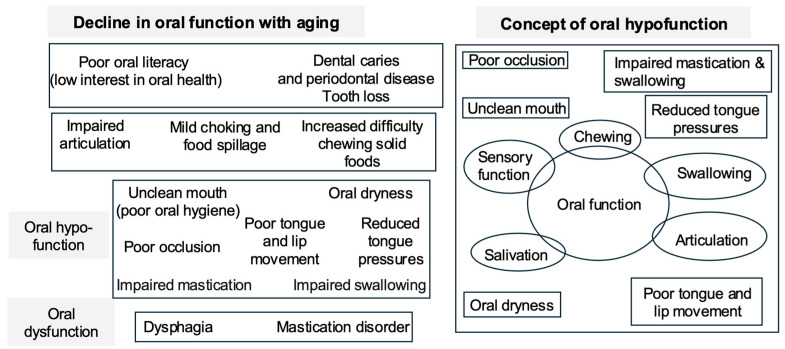
Concept of oral hypofunction as defined by the Japanese Society of Gerodontology. This figure illustrates the relationship between age-related decline in oral function and the conceptual framework of oral hypofunction. Progressive deterioration in oral conditions—such as dental caries, periodontal disease, tooth loss, reduced oral hygiene, and decreased oral literacy—may lead to impairments across multiple functional domains, including mastication, swallowing, tongue–lip motor function, tongue pressure, salivation, and occlusion. Oral hypofunction can be understood as an intermediate state between age-related decline in oral function and overt oral dysfunction, such as dysphagia and mastication disorders. This model emphasizes that oral hypofunction is a multifactorial and potentially reversible condition with appropriate assessment and intervention. Concept adapted from [46].

Aligned with these policy developments and in response to unprecedented population aging, the concept of oral health in Japan has expanded beyond conventional dental treatment—such as caries control and restoration of masticatory function—to encompass oral hygiene practices and functional interventions directly related to dysphagia rehabilitation [46] (Figure 5). These include structured swallowing therapy, compensatory postural strategies, and targeted oropharyngeal exercises. In 2018, “oral hypofunction” was formally recognized in Japan as a reimbursable clinical condition under the dental insurance system, referring to a clinically defined decline in multiple oral functions—such as mastication, salivation, tongue–lip motor function, and swallowing—rather than impairment in a single domain [42,68] (Figure 6). This concept reflects the cumulative and interrelated nature of oral functional deterioration in older adults and its close association with dysphagia and frailty. Pediatric oral function management was also incorporated into dental disease management fees [68]. Collectively, these reforms institutionalized the involvement of dental professionals in dysphagia prevention and rehabilitation.

**Figure 7:**
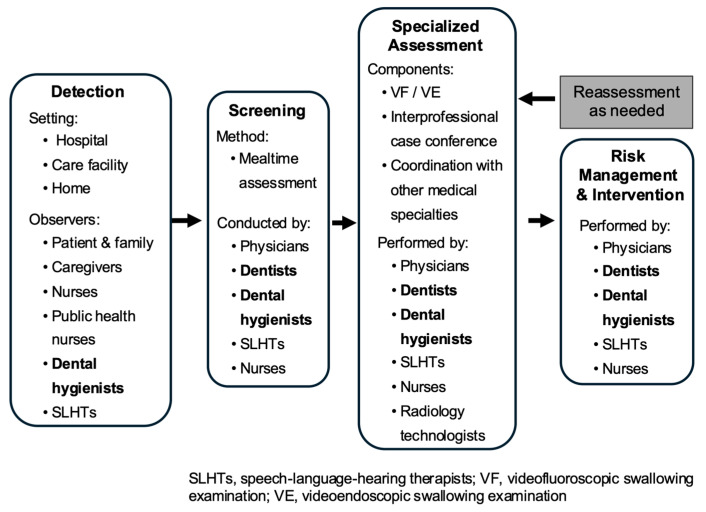
Clinical workflow of dysphagia rehabilitation in Japan. This figure presents a typical clinical workflow of dysphagia rehabilitation in Japan, illustrating the sequential processes from detection and screening to specialized assessment and risk management, with reassessment conducted as needed. Interprofessional collaboration is emphasized throughout the workflow. Dental professionals, including dentists and dental hygienists, participate across all stages of care, contributing to oral function assessment and the support of safe eating and swallowing within team-based care. Grey shading indicates reassessment processes performed as needed, and bold text highlights dental professionals to emphasize their role in dysphagia management.

Building on these institutional and conceptual developments, dental professionals have been systematically integrated into the clinical workflow of dysphagia rehabilitation (Figure 7). Their roles span screening, assessment, intervention, and long-term follow-up. Dental hygienists participate at every stage—from early screening to maintenance in long-term care settings—working closely with physicians, SLHTs, dietitians, and nursing staff. Dentists, alongside physicians, assume key clinical responsibilities, including diagnostic evaluation, participation in interdisciplinary case conferences, and delivery of direct clinical interventions, such as swallowing-related oral function therapy, perioperative oral management, and prosthetic fabrication. Together, dental professionals function as a critical bridge, facilitating collaborative practice and continuity of care across all phases of dysphagia management.

In outpatient dental settings, dysphagia-related care typically includes screening tests such as the Repetitive Saliva Swallowing Test (RSST), Modified Water Swallowing Test (MWST), food tests, cervical auscultation, and the Eating Assessment Tool-10 (EAT-10). Clinical management further involves assessment and treatment of oral hypofunction and mastication disorders; nutritional evaluation; oral hygiene management; and prosthetic interventions, including obturators, palatal augmentation plates, palatal lift prostheses, and speech aids. Depending on clinical indications, dentists may also conduct instrumental swallowing assessments (VE/VF examination), provide indirect and direct swallowing training, implement postural strategies and diet modification based on clinical swallowing and mealtime assessments, deliver perioperative oral care, and educate patients and caregivers.

Because of equipment limitations, home-visit dental care focuses primarily on bedside assessment, essential oral care, and basic dental treatments such as simple extractions, composite restorations, and minor denture repairs. In home-care settings, dentists commonly perform VE examination, enabling timely evaluation, individualized intervention, and caregiver education within patients’ daily living environments.

However, the extent of dental involvement may vary across settings, and issues related to role standardization and interprofessional boundaries remain under discussion.

2. Secondary Medical Care Facilities

Secondary medical care facilities occupy an intermediate position within Japan’s medical care system, managing conditions that exceed the scope of individual clinics, including cases requiring hospitalization, emergency response, or coordinated inpatient care [64]. In the context of dysphagia rehabilitation, these facilities typically manage patients with moderate to severe swallowing disorders who require inpatient monitoring, interprofessional intervention, or escalation of care beyond outpatient settings.

Within the regional care network, secondary facilities function as essential intermediaries, providing 24 h medical coverage and serving as a clinical safety net for community-based and home medical services. Their responsibilities include coordination with visiting physicians, nursing stations, visiting dental services, and medication management, thereby supporting continuity of care beyond hospital settings.

Secondary facilities are broadly categorized according to bed capacity:

- Small and medium-sized hospitals (< 200 beds): These hospitals primarily serve local communities and maintain close collaboration with nearby clinics. Home-visit medical services are generally limited to a defined geographic radius (approximately 16 km), although exceptions may be granted based on regional needs.
- Regional medical support hospitals (≥ 200 beds): These institutions mainly accept referred patients and provide more specialized inpatient and outpatient services. Although their direct involvement in home medical care is limited, they play a pivotal coordinating role within regional referral networks.

Collectively, secondary medical care facilities function as central hubs linking primary clinics, tertiary hospitals, and community-based services, thereby facilitating seamless transitions and continuity in dysphagia rehabilitation across care settings.

3. Tertiary Medical Care Facilities

Tertiary medical care facilities are designed to deliver highly specialized interventions for patients requiring advanced medical resources, including those in acute phases or with complex comorbidities [64]. In the context of dysphagia, this includes patients with severe swallowing disorders requiring advanced diagnostic evaluation, intensive interprofessional management, or surgical intervention.

- Special function hospitals (≥400 beds): These institutions are equipped with advanced diagnostic technologies and multidisciplinary specialist teams. In dysphagia care, they are responsible for comprehensive assessment and management of severe conditions, including aspiration prevention surgeries.

Unlike primary and most secondary facilities, tertiary hospitals generally do not provide home-visit medical or dental services. Their role is concentrated on acute intervention and advanced treatment, followed by coordinated transfer of patients to secondary facilities, primary clinics, or long-term care services once clinical stability has been achieved.

Beyond facilities operating under the UHS, long-term care institutions covered by the LTCIS function as downstream care settings within Japan’s dysphagia care pathway. These facilities primarily accommodate patients with chronic or persistent swallowing disorders who require ongoing rehabilitation, daily risk management, and long-term functional support beyond the acute medical phase. Their role is to sustain swallowing-related function and safety over time, thereby complementing hospital- and clinic-based medical services.

### 4.3. Healthcare Insurance Systems and the Community-Based Integrated Care System (CBICS)

While the roles of medical, dental, and long-term care facilities define the structural framework of dysphagia rehabilitation, the functional integration of these services in Japan is fundamentally supported by its healthcare insurance systems and community-based care policies. In particular, the UHS, the LTCIS, and the CBICS jointly ensure continuity of dysphagia management across acute, convalescent, and chronic phases.

#### 4.3.1. The UHS and LTCIS

Japan’s UHS, established in 1961, provides comprehensive medical coverage for all citizens and is characterized by its “free access” principle, which allows patients to seek care at medical institutions of their choice [63]. Under this system, dysphagia-related care is delivered through inpatient, outpatient, and home-visit medical services. Home-visit medical care, in particular, is recognized as a distinct modality, supporting patients during the convalescent phase after acute treatment as well as those with chronic conditions requiring ongoing management. By enabling care delivery in patients’ living environments, this approach also helps reduce reliance on hospital-based services.

Complementing the UHS, the LTCIS was introduced in 2000 to distribute responsibility for elderly care across society and alleviate the burden on families [63]. The LTCIS covers individuals aged 40 years and older and finances a wide range of services, including facility-based care, visiting nursing, day services, and long-term rehabilitation. Visiting nursing services—provided mainly by hospitals, clinics, and nursing stations—may be delivered under either the UHS or the LTCIS, depending on patients’ medical conditions and functional needs.

A defining feature of the LTCIS is the role of care managers, who are long-term care support specialists responsible for developing individualized care plans. Acting as coordinators, care managers integrate medical treatment, rehabilitation, nursing care, and welfare services while mediating among municipalities, service providers, and care facilities. This coordinating function is particularly critical for patients with dysphagia, who often require sustained, interprofessional support beyond the acute phase.

Collectively, the UHS and LTCIS provide comprehensive coverage for dysphagia rehabilitation across the care continuum. Following hospital discharge, patients may receive facility-based or home-based rehabilitation under the LTCIS or continue medical management through home-visit services financed by the UHS. To further strengthen continuity of care, the UHS allows additional reimbursement for hospitals that utilize information and communication technology (ICT) systems to share patient information with LTCIS-affiliated rehabilitation and care providers, thereby enhancing safety and coordination during transitions between medical and long-term care sectors [69].

#### 4.3.2. The CBICS

Building upon the parallel operation of the UHS and LTCIS, Japan began developing the CBICS in the early 2000s, with the strategic goal of establishing a locally integrated care framework nationwide by 2025 [70]. The CBICS is designed to ensure that essential services—including medical care, long-term care, preventive services, housing, and livelihood support—are accessible within a defined daily living area, often conceptualized as a community unit roughly corresponding to a junior high school district, where key services can typically be reached within about 30 min [70].

Within this framework, the CBICS functions as an institutional platform for integrating medical services covered by the UHS with long-term care services financed by the LTCIS. In the context of dysphagia rehabilitation, the CBICS promotes structured collaboration among clinics, hospitals, dental facilities, rehabilitation providers, home-visit services, and care managers, thereby enabling seamless care delivery across care settings and disease stages.

### 4.4. Vertical and Horizontal Collaboration

**Figure 8:**
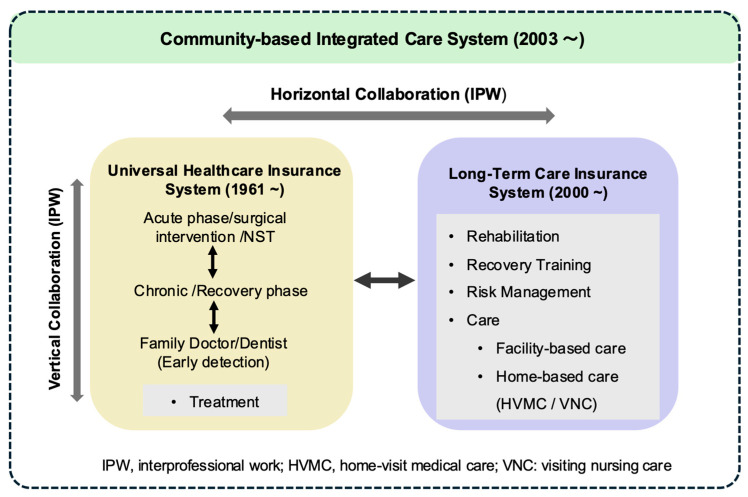
Conceptual framework of dysphagia rehabilitation practice within Japan’s healthcare system. This figure illustrates the structural framework of dysphagia rehabilitation across Japan’s UHS, LTCIS, and CBICS. In this framework, vertical collaboration reflects continuity of care across disease phases within the medical care system, while horizontal collaboration represents coordination between medical and long-term care services. IPW operates throughout this framework as a collaborative mechanism within medical, dental, rehabilitation, and community care providers, among others, supporting the integration of acute, recovery, and community-based care for comprehensive management of dysphagia. Background colors are used to distinguish between the healthcare and long-term care insurance systems.

Within the interconnected frameworks of the UHS, the LTCIS, and the CBICS, Japan’s dysphagia rehabilitation model is structured around both vertical and horizontal collaboration to ensure timely transitions, appropriate allocation of resources, and continuity of management across the disease trajectory. Vertical collaboration governs transitions across levels of medical care, whereas horizontal collaboration facilitates coordination between the medical and long-term care sectors (Figure 8).

#### 4.4.1. Vertical Collaboration Across Levels of Medical Care

Vertical collaboration refers to coordination among primary, secondary, and tertiary care facilities according to patients’ clinical severity and rehabilitation needs. In Japan, although patients are generally allowed to access medical institutions freely under the UHS, this process is facilitated by a formal referral letter system, which standardizes information exchange and supports orderly dysphagia-related referrals between institutions.

Patients requiring advanced diagnostic evaluation, surgical intervention, or intensive acute management are referred from primary or secondary care settings to tertiary special-function hospitals. These tertiary institutions provide highly specialized assessments and treatments during the acute phase. Once clinical stability is achieved or acute interventions are completed, patients who require ongoing rehabilitation, nutritional management, or longitudinal monitoring are systematically transferred back to secondary or primary care facilities that are better suited for convalescent and rehabilitative care (Figure 8).

This bidirectional referral structure enables patients to move efficiently across levels of care as their conditions evolve. By aligning care intensity with clinical need, vertical collaboration ensures that highly specialized medical resources are reserved for complex cases, while rehabilitation and long-term management are delivered in lower-acuity, more accessible settings.

#### 4.4.2. Horizontal Collaboration Between Medical and Long-Term Care Sectors

Horizontal collaboration addresses coordination between medical institutions operating under the UHS and long-term care facilities supported by the LTCIS. As Japan’s healthcare system increasingly emphasizes community-based integrated care, such cross-sector collaboration has become essential for managing chronic dysphagia and reducing avoidable hospital utilization.

Following discharge from medical institutions, patients in the post-acute or chronic phase who require prolonged rehabilitation, nutritional support, or daily risk management are commonly transitioned to LTCIS-based long-term care facilities or home-care services. Conversely, residents of long-term care facilities who experience medical deterioration, aspiration-related complications, or acute illness are promptly referred back to appropriate UHS medical institutions for diagnostic evaluation and treatment (Figure 8).

Through this continuous bidirectional exchange between medical and long-term care sectors, horizontal collaboration supports uninterrupted dysphagia management across care settings, reduces delays in intervention, and improves the efficiency of community-level healthcare resource allocation. Together, these vertical and horizontal coordination mechanisms form the operational backbone of Japan’s dysphagia rehabilitation model, enabling integrated management across medical and long-term care settings.

### 4.5. Interprofessional Work (IPW) in Dysphagia Rehabilitation in Japan

While the UHS, LTCIS, and CBICS provide the structural foundation for dysphagia care coordination, effective implementation of these collaborative mechanisms ultimately depends on interprofessional work (IPW). The management of dysphagia requires coordinated attention to multiple interrelated domains, including swallowing physiology, nutrition and hydration, oral hygiene, and coexisting medical conditions. Given this inherent complexity, effective dysphagia care cannot be adequately delivered by a single profession and instead relies fundamentally on interprofessional work (IPW). In this review, IPW refers broadly to collaborative practice among multiple healthcare professions involved in dysphagia management, encompassing interdisciplinary and transdisciplinary forms of teamwork depending on the clinical setting.

In Japan, core IPW teams for dysphagia rehabilitation typically include physicians (e.g., rehabilitation specialists, neurologists, and otorhinolaryngologists), dentists, speech–language–hearing therapists (SLHTs), registered nurses (RNs), and dental hygienists (DHs). Depending on patient characteristics and care settings, additional professionals—such as registered dietitians (RDs), occupational therapists (OTs), physical therapists (PTs), pharmacists, and palliative-care specialists—may also be involved. Importantly, patients and their families are regarded as integral members of the care team, particularly in community- and home-based settings, with social workers and home caregivers participating as needed. Together, these professionals form flexible, setting-specific teams that adapt to disease severity, care environment, and available resources.

#### 4.5.1. Distinctive Features of Japan’s IPW Framework

Japan’s IPW framework for dysphagia rehabilitation is characterized by several distinctive professional roles and credentialing systems that support advanced, coordinated care across settings:

- Consistent with the roles described above, dentists in Japan are actively involved in VF and VE examinations in clinical practice, reflecting their recognized role in swallowing assessment, particularly in the preparatory and oral phases;
- SLHTs, under the supervision of physicians or dentists, are the only professionals authorized to conduct medical swallowing rehabilitation. They are also certified to perform sputum suction, a responsibility rarely assigned to SLHTs internationally [71]. Notably, close collaboration between dentists and SLHTs has been a defining feature of Japanese dysphagia rehabilitation since the establishment of the JSDR, particularly in home-visit medical and dental care, where joint bedside assessments and coordinated interventions are essential.
- Certified dysphagia nurses and advanced dental hygienists represent another unique aspect of the Japanese system. Japan appears to be one of the few countries with formal national-level certification programs in dysphagia rehabilitation for both RNs and DHs. Certified dysphagia nursing was established in 2005 under the Japanese Nursing Association’s Certified Nurse system. According to certification statistics published by the Japanese Nursing Association, more than 1000 registered Certified Nurses in dysphagia nursing were active nationwide as of December 2024, indicating sustained professional engagement across hospital, long-term care, and community-based practice settings [72]. In parallel, the Japan Dental Hygienists’ Association launched the Certified Dental Hygienist System in 2008, providing advanced training in dysphagia-related oral care and rehabilitation [24].

Collectively, these credentialing systems support the delivery of high-quality, specialized IPW across the continuum of dysphagia care.

#### 4.5.2. Transdisciplinary Collaboration in Dysphagia Care

**Figure 9:**
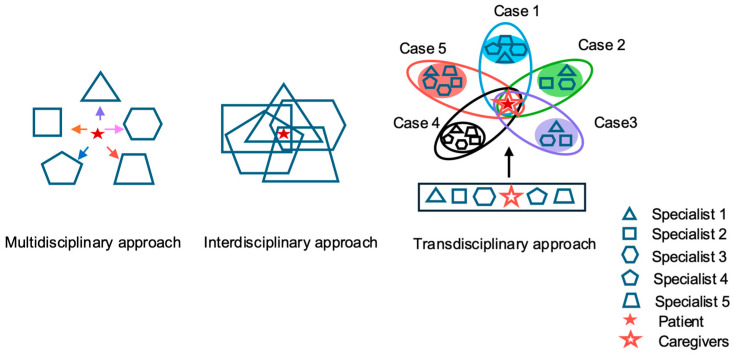
Comparison of multidisciplinary, interdisciplinary, and transdisciplinary approaches in dysphagia rehabilitation. In the multidisciplinary approach, specialists from different disciplines work in parallel with limited interaction, each maintaining distinct professional roles centered on the patient. In the interdisciplinary approach, partial overlap and coordination among disciplines occur through shared goals and communication, while professional boundaries remain identifiable. In contrast, the transdisciplinary approach emphasizes dynamic role integration and flexible collaboration, in which professional roles and skills are shared across disciplines and care is reorganized around patient-centered needs. Caregivers are regarded as integral members of the care team. Cases 1–5 illustrate example scenarios showing how team configurations may vary according to clinical contexts within a transdisciplinary framework. Shapes represent different professional disciplines. The filled star indicates the patient, and the open star indicates caregivers. Colors, arrows, and enclosed shapes (circles and ellipses) are used to illustrate different interaction patterns, case groupings, and the integration of disciplines across approaches.

Beyond multidisciplinary and interdisciplinary models, Japan has increasingly adopted a transdisciplinary approach to IPW in dysphagia rehabilitation. In this approach, professionals not only share treatment goals but also assume overlapping and complementary responsibilities, dynamically adjusting their roles in response to patient needs, disease severity, and care environments (Figure 9). Such transdisciplinary teamwork is characterized by flexible role boundaries and dynamic role integration. This approach has been described as a common and pragmatic approach in Japanese dysphagia rehabilitation, particularly in community-based and home care, which are characterized by comparatively limited professional resources and diverse patient needs [73,74].

Representative examples of transdisciplinary collaboration include:

- Joint meal observation and monitoring of swallowing safety by RNs and DHs;
- Collaborative evaluation of swallowing function and oral care strategies by physicians, dentists, SLHTs, RNs, and DHs;
- Close coordination between physicians or dentists and RNs and RDs in determining oral intake plans and managing complications;
- Coordinated education and guidance provided by multiple team members to patients, families, and caregivers.

This flexible role-sharing offers several advantages:

1. Efficient decision making through shared clinical perspectives;
2. Tailored team composition, ensuring that only necessary professionals are involved (e.g., SLHTs or RDs may not be required in mild cases); and
3. Patient- and family-centered care, in which trained non-professional caregivers can safely undertake delegated tasks—such as oral care, meal observation, or basic sputum suction following appropriate training and authorization—under professional supervision.

Such adaptability enhances system resilience and continuity of care in aging communities with increasingly complex and evolving needs.

**Figure 10:**
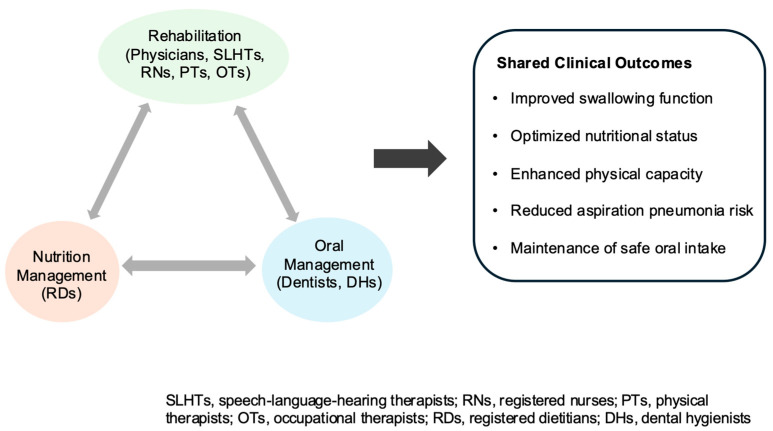
Triadic collaborative care framework applied in dysphagia management. This figure illustrates a nationally promoted collaborative care framework in Japan that integrates rehabilitation, nutrition support, and oral management, applied in the care of older adults, including those with dysphagia. Rehabilitation involves physicians, nurses, SLHTs, PTs, and OTs; nutrition management is provided by RDs; and oral management is delivered by dentists and DHs. Bidirectional arrows indicate reciprocal collaborative interactions and shared clinical decision making among the three domains. Together, this integrated triad supports the maintenance and improvement of swallowing function, optimization of nutritional intake, a reduction in aspiration pneumonia risk, and continued oral feeding. Background colors are used to distinguish different care domains.

These interprofessional and transdisciplinary practices are further reflected in a triadic collaborative framework that integrates swallowing rehabilitation, nutritional management, and oral health management in dysphagia care (Figure 10).

#### 4.5.3. National Promotion of the “Rehabilitation–Nutrition–Oral Management Triad”

Collaboration among rehabilitation, nutrition support, and oral management is particularly critical in dysphagia care, where impairments in swallowing, nutritional status, and oral function are closely interrelated. Existing evidence suggests that improved oral hygiene and structured oral management are associated with a reduced incidence of aspiration pneumonia and related mortality in frail older adults [75]. In parallel, effective swallowing rehabilitation facilitates oral intake and enhances the effectiveness of nutritional interventions, while adequate nutritional status is considered essential for maintaining muscle function and overall functional capacity [76].

Despite its clear clinical relevance, systematic collaboration across these three domains remained underdeveloped in Japan for many years [21]. In response to the growing demands of population aging and the recognition of sarcopenic dysphagia as a multifactorial condition, the concept of a “rehabilitation–nutrition–oral management triad” (Figure 10) has been proposed as an integrative framework to address these interconnected challenges (Figure 10) [21]. Reflecting its policy relevance, this framework has been incorporated into national policy initiatives, including the Basic Policy on Economic and Fiscal Management and Reform and the 2024 medical fee revision [49,77].

Emerging clinical evidence provide preliminary support for this integrative approach. Combined interventions involving rehabilitation, nutrition support, and oral management have been associated with improvements in activities of daily living (ADLs) and muscle-related outcomes among hospitalized patients with stroke and dysphagia [78]. In addition, observational evidence suggests that the involvement of registered dietitians and dental hygienists within interprofessional teams may contribute to improved functional outcomes [22]. This integrative approach is conceptually supported by the reciprocal relationship between muscle function, nutritional status, and oral health in the pathophysiology of dysphagia.

However, it should be noted that the triad model is a relatively recent conceptual development, and the current evidence base remains limited in scale and methodological rigor, consisting primarily of observational and single-center studies.

Therefore, while the available findings suggest the potential clinical value of integrating rehabilitation, nutrition, and oral management, these findings should be interpreted as preliminary and context-dependent, and further high-quality studies are required to establish its effectiveness and to quantify its impact on both clinical outcomes and healthcare system performance.

#### 4.5.4. Workforce Structure and Interprofessional Capacity in Japan

The implementation of the rehabilitation–nutrition–oral management triad is closely linked to workforce composition and interprofessional capacity. Japan has a relatively well-developed infrastructure of healthcare professionals involved in dysphagia care, including rehabilitation therapists, registered dietitians, and dental professionals. In particular, the integration of dental hygienists and dietitians into hospital-based and community-based care teams has been increasingly emphasized in recent years.

However, current evidence remains insufficient to quantitatively establish the relationship between workforce density and patient outcomes in this field. Therefore, while the availability of relevant professionals may facilitate the delivery of integrated care, its direct impact on clinical effectiveness has yet to be clearly demonstrated.

#### 4.5.5. Implications for Healthcare Burden and System Efficiency

From a health systems perspective, the integration of rehabilitation, nutrition, and oral management is conceptually associated with potential reductions in healthcare burden by lowering complication rates and supporting functional recovery. For example, improved oral management has been associated with a lower incidence of aspiration pneumonia, a major contributor to hospitalization and mortality among older adults [75]. In addition, interventions targeting functional recovery and nutritional status may support earlier rehabilitation gains and reduce the risk of functional decline.

However, direct evidence linking such integrated care approaches to reductions in healthcare utilization—such as length of hospital stay, readmission rates, or medical costs—remains limited. Therefore, while such integrated care approaches are conceptually aligned with strategies to enhance system efficiency in aging societies, their impact at the healthcare system level has yet to be fully quantified. Further evaluation using system-level indicators is warranted.

#### 4.5.6. Pediatric IPW in Dysphagia Care

Although this review primarily focuses on dysphagia rehabilitation in older adults, pediatric dysphagia care demonstrates the broader application of IPW. In pediatric settings, interprofessional collaboration extends beyond healthcare to include educators, disability-support staff, and welfare service providers. For school-aged children, the IPW team often involves parents, teachers, and staff from special-support schools or residential facilities. In Japan, pediatric dysphagia management increasingly requires coordination across healthcare, education, welfare, and family–community sectors, consistent with broader national policy trends promoting cross-sector support for children with special health care needs [79,80]. This pediatric framework highlights the adaptability of Japan’s IPW framework across age groups and care systems, reinforcing the broader relevance of transdisciplinary and cross-sector approaches beyond geriatric dysphagia care.

### 4.6. Challenges and Limitations of the Japanese Dysphagia Rehabilitation Model

Despite its advanced development, the Japanese model of dysphagia rehabilitation presents several important structural and practical limitations that warrant careful critical consideration.

First, regional disparities remain a structural concern. While urban areas benefit from well-established multidisciplinary teams, including access to dental professionals, service availability in rural and resource-limited settings is comparatively constrained. This imbalance is partly attributable to the urban concentration of dental clinics and workforce, a well-documented feature of Japan’s healthcare landscape, which may lead to inequities in access to comprehensive dysphagia care and limit nationwide consistency in service delivery.

Second, although the integration of dental professionals represents a distinctive strength of the Japanese model, it also introduces challenges related to workforce capacity and training standardization. Expanding such roles requires sustained investment in interdisciplinary education, clear competency frameworks, and institutional coordination. In practice, variability in training opportunities and coordination structures across institutions may hinder the uniform implementation of this model.

Third, questions regarding cost-effectiveness and long-term sustainability remain insufficiently addressed. The resource-intensive nature of comprehensive, team-based care may increase short-term healthcare utilization, yet robust economic evaluations examining cost–benefit balance and long-term system efficiency are still limited. This represents a critical gap, particularly in the context of Japan’s rapidly aging population and rising healthcare expenditure, where efficiency and sustainability are of increasing importance. This gap may limit the ability of policymakers to assess the scalability and long-term viability of the model.

Fourth, the transferability of the Japanese model to other countries is not straightforward. Its implementation is supported by specific policy frameworks, reimbursement systems, and professional norms—particularly the institutionalized role of dentistry within medical care—that are not universally present. As such, direct adoption in other healthcare systems may face significant structural and cultural barriers.

Fifth, although this review highlights the organizational and conceptual strengths of the Japanese system, the evidence base supporting clinical effectiveness remains heterogeneous. In particular, high-quality studies directly evaluating the impact of dental involvement and team-based care on patient-centered outcomes—such as aspiration pneumonia incidence, nutritional status, and quality of life—are still limited. This constrains the ability to draw definitive conclusions regarding the effectiveness of the model and underscores the need for more rigorous empirical research.

These limitations suggest that the Japanese model should be interpreted within its specific systemic and cultural context.

At the professional level, although dental professionals play an important role within interprofessional teams in Japan’s dysphagia rehabilitation system, several profession-specific challenges and ongoing debates should be acknowledged.

The extent of dental involvement in swallowing assessment and management varies across institutions and regions, depending on the availability of trained personnel, equipment, and local coordination structures. This variability may lead to heterogeneity in clinical practice and service delivery.

Furthermore, the expanded role of dentists in procedures such as VE and VF examinations, while it is increasingly recognized within Japanese clinical and policy contexts, is not widely adopted internationally. This raises questions regarding the transferability of this model to healthcare systems with different regulatory frameworks and professional boundaries.

Moreover, the evolving scope of dental practice in dysphagia care has generated ongoing discussions regarding role delineation, competency standardization, and potential overlap with other professionals, particularly speech–language–hearing therapists and physicians.

Taken together, despite strong policy support, the evidence base evaluating the effectiveness and cost-efficiency of dentist-integrated dysphagia interventions remains limited. These profession-specific challenges reflect broader system-level issues related to standardization, workforce development, and cross-disciplinary coordination, underscoring the need for further high-quality research.

### 4.7. International Context and Comparative Perspective

While this review does not aim to provide a comprehensive cross-national comparison, it is important to situate the Japanese dysphagia rehabilitation model within a broader international context.

In many Western healthcare systems, particularly in North America, dysphagia management is typically led by speech–language pathologists (SLPs), with comparatively limited routine involvement of dental professionals. In contrast, the Japanese model is characterized by the early and systematic integration of dental professionals, particularly in oral management and the prevention of aspiration-related complications. This distinction reflects broader systemic differences in professional scope of practice, healthcare policy frameworks, and the institutional organization of team-based care. In addition, interprofessional collaboration in many Western settings is often structured in a more discipline-specific or hierarchical manner, with clearly delineated roles and less overlap between professions. By contrast, the Japanese model more frequently adopts an integrated or transdisciplinary approach, in which role boundaries are more flexible and responsibilities may be shared across professionals depending on clinical context.

From a benchmarking perspective, these differences help clarify the distinctive features of the Japanese model within the global landscape of dysphagia care and highlight which elements may be context-specific versus potentially adaptable to other healthcare systems.

Furthermore, Japan’s approach places strong emphasis on system-level coordination across acute care, long-term care, and community-based settings. While integrated care models are increasingly promoted internationally, the degree of formalized collaboration observed in Japan—supported by national policies and reimbursement structures—remains relatively distinctive.

These differences do not imply that the Japanese model is directly transferable to other countries. Rather, they highlight how historical, institutional, and cultural factors shape the development of dysphagia rehabilitation systems. In this context, the Japanese experience may provide contextual insights, while requiring careful adaptation to local healthcare structures. This perspective underscores the importance of context-sensitive implementation when considering the translation of complex care models across health systems.

## 5. Implementation Challenges and Future Directions

Despite substantial progress over the past four decades, Japan’s dysphagia rehabilitation system continues to face several structural and operational challenges that affect its long-term sustainability and adaptability. These challenges arise from the complexity of healthcare financing, limitations in national data infrastructure, and regional disparities in healthcare resources. Such issues have become increasingly salient in the context of rapid population aging and expanding community-based care.

### 5.1. System Complexity Arising from Dual Reimbursement Frameworks

The coexistence of the UHS and the LTCIS creates overlapping reimbursement frameworks, particularly in home-based dysphagia management. Procedures such as oral cleaning or oral functional management may be reimbursed under either system; however, LTCIS often takes precedence in home-care settings, which can create confusion regarding appropriate billing pathways. This duality generates administrative complexity at the policy–practice interface, increases the risk of billing inconsistencies, and places additional burdens on both clinicians and care coordinators. Such fragmentation can constrain clinical efficiency and impede smooth coordination of care in community and home-care settings.

### 5.2. Insufficient National Data for Evidence-Based Planning

As visiting medical and dental services continue to expand, the absence of a unified national database poses a major limitation for evidence-based policy development, quality monitoring, and workforce planning. Comprehensive data remain incomplete across regions and care settings, particularly with respect to facility capacity, workforce distribution, training availability, and operational challenges. The COVID-19 pandemic further disrupted national surveys and routine audits, widening information gaps at a time when accurate epidemiological and service data were urgently needed to guide planning within the CBICS and resource allocation.

### 5.3. Regional Disparities and the Need for Adaptable Care Models

Japan exhibits substantial regional variation in demographic structure and healthcare resources. Large metropolitan areas face hospital congestion, workforce shortages, and growing demand for home-based dysphagia services driven by high population density and constrained healthcare capacity. In contrast, rural and depopulated regions often rely more heavily on centralized, facility-based dysphagia care due to limited personnel and infrastructure. These contrasting realities underscore the limitations of a uniform national care model and highlight the necessity of region-specific care frameworks that accommodate local demographics, resources, and service needs. Future policy design will therefore need to balance national standardization with local flexibility.

### 5.4. Strengthening Care Coordination and Information Sharing

Although interprofessional work (IPW) is strongly promoted in national policy, practical barriers persist, including inconsistent communication, fragmented documentation, and limited information sharing between medical and long-term care providers. Digital health platforms represent a promising partial response to these challenges. For example, Hakodate City’s “ID-Link” system enables secure sharing of prescriptions, laboratory data, and imaging information across healthcare and welfare institutions, facilitating continuity of care [81]. Similar platforms are now being introduced nationwide, particularly to support home-based dysphagia management.

In parallel with digital integration, workforce development initiatives are also expanding. Dysphagia-related competencies are now incorporated into national curricula across medicine, dentistry, nursing, nutrition, and dental hygiene, and are reflected in national licensing examinations. In addition, clinical training in home-based care has become mandatory for physicians and dentists, aligning professional preparation with the increasing shift toward community-based practice.

Continuing education mechanisms further support standardization of skills, including e-learning programs and certification systems administered by the JSDR. Regional re-education centers also contribute to workforce capacity building. For example, Kyushu Dental University’s Dental Center for the Medically Compromised Patient (DEMCOP), established in 2016, was among the first facilities in Japan dedicated to structured retraining of young dentists in elder care, dysphagia management, and emergency response. Together, these educational and training structures constitute an institutional foundation for interprofessional dysphagia care, although broader systemic coordination remains an ongoing policy challenge. These challenges are particularly relevant for dental professionals, whose expanding roles in dysphagia care depend on well-coordinated interprofessional frameworks, consistent training standards, and effective information-sharing mechanisms across care settings.

### 5.5. Preparedness for Pandemics and Natural Disasters

The COVID-19 pandemic exposed systemic vulnerabilities in dysphagia care, particularly the reliance on aerosol-generating gold-standard assessments such as VF and VE examinations. Infection control requirements limited the safe implementation of these procedures, disrupting diagnostic processes and delaying clinical decision making. To enhance preparedness for future pandemics and natural disasters, Japan will need to develop non-contact or low-contact assessment alternatives, establish clear risk stratification protocols, and design flexible care pathways suitable for crisis conditions. Training clinicians in telehealth-assisted evaluations and crisis-adapted dysphagia management will be essential to ensuring continuity of care for highly vulnerable older populations.

Addressing these challenges will be essential for ensuring the long-term sustainability, equity, and resilience of Japan’s dysphagia rehabilitation system as population aging and community-based care continue to expand.

## 6. Limitations

This narrative review focuses specifically on Japan’s dysphagia rehabilitation system and does not aim to provide direct international comparisons. Although representative studies, policy documents, and academic society guidelines were included, the narrative approach does not constitute a systematic or quantitative synthesis of evidence. In addition, pediatric dysphagia care is discussed only briefly, reflecting the primary emphasis on adult and geriatric populations in alignment with Japan’s demographic profile. Finally, reliance on Japanese-language sources may limit accessibility for international readers and constrain broader contextual interpretation.

## 7. Conclusions

Japan’s dysphagia rehabilitation system has evolved in parallel with the country’s demographic transitions, resulting in a mature and interconnected care model spanning acute hospitals, rehabilitation facilities, long-term care institutions, and community- and home-based care. A defining feature of this system is the active involvement of dental professionals, who contribute to swallowing assessment, oral management, oral functional rehabilitation, and interprofessional care planning and decision making across care settings. Their expanding roles—together with those of SLHTs, rehabilitation physicians, dietitians, nurses, and care managers—reflect Japan’s sustained emphasis on transdisciplinary collaboration.

As global population aging accelerates, Japan’s experience offers valuable insights into how coordinated policy frameworks, interprofessional care, and the integration of oral management into rehabilitation pathways can support the development of safe, efficient, and sustainable dysphagia care systems. Continued efforts to simplify system operations and strengthen IPW will be essential to maintaining the resilience of this system.

At the same time, addressing regional disparities and enhancing preparedness for public health emergencies and natural disasters remain critical future priorities. While Japan’s dysphagia rehabilitation system is shaped by a unique institutional context, its underlying principles provide a meaningful reference for other aging societies seeking to strengthen dysphagia care systems.

Notwithstanding these contributions, several limitations of this review should also be acknowledged. As a narrative review, this study does not provide a systematic or quantitative synthesis of evidence, and the selection of literature may be subject to interpretive bias. Furthermore, the analysis primarily focuses on the Japanese context, which may limit the generalizability of the findings to other healthcare systems.

Future research is needed to strengthen the evidence base for dysphagia rehabilitation models, particularly through high-quality comparative studies, longitudinal outcome evaluations, and economic analyses examining cost-effectiveness and system efficiency. In addition, further investigation into the role of dental professionals and collaborative care models across different healthcare systems would help clarify the transferability and scalability of integrated dysphagia care models.

## Data Availability

No new data were created or analyzed in this study.

## Abbreviations

The following abbreviations are used in this manuscript:

QoL	Quality of life
WHO	World Health Organization
JSDR	Japanese Society of Dysphagia Rehabilitation
IPW	Interprofessional work
LTCIS	Long-term care insurance system
CBICS	Community-based integrated care system
HCSCs	Home care support clinics/hospitals
NSTs	Nutrition support teams
VE	Videoendoscopic swallowing examination
VF	Videofluoroscopic swallowing examination
UHS	Universal healthcare system
RSST	Repetitive saliva swallowing test
MWST	Modified water swallow test
MHLW	Ministry of Health, Labour and Welfare
SLHTs	Speech–language–hearing therapists
SLPs	Speech–language pathologists
RNs	Registered nurses
DHs	Dental hygienists
RDs	Registered dietitians
OTs	Occupational therapists
PTs	Physical therapists
ADLs	Activities of daily living
COVID-19	Coronavirus disease 2019
DEMCOP	Dental center for the medically compromised patient
